# The Coming of Easter

**Published:** 1896-03-28

**Authors:** 


### The Coming of Easter.

The approach of Easter reminds us again of the nuisance to all concerned which is involved in the uncertain time of its arrival. It is not too much to say that the health of thousands, and even the life of no small number of people, depends on the date on which Easter may happen to fall. For not only is Easter involved, but Whitsuntide and even that great English festival the Derby Day; and so it happens that what are now great national holidays, for the due and healthy enjoyment of which warm weather is of the first importance, are fixed by an ancient formula and in accordance with exigencies of moons, calendars, and golden numbers, which have no relation to the conditions of affairs at the present time.

That the Easter festival is used by the Christian Churches as a memento and celebration of certain great and serious events in the history and inception of their religion is a matter which would be by no means interfered with by the time of its recurrence being fixed, instead of being allowed to wander over the interval between March 22nd and April 25th. No one pretends that December 25th is the true date of the birth of Christ, yet that is the meaning of Christmas Day, a festival which is none the less carefully kept from the fact of its chronological inaccuracy. As for Easter, even the day on which it was to be celebrated, *i.e.*, whether it was to be on a Sunday or on the Passover of the Jews, remained a matter of dispute for the first three hundred years of the Christian era; and when in the end it was decided to celebrate the resurrection of Christ upon a Sunday the artificiality of the arrangement was established, for it can only occasionally happen that the Sunday can fall on the proper day.

Not that we imagine that the Easter of our Churches will ever be altered; but no one who has watched modern developments can pretend that Bank Holidays are religious festivals, and it is every year becoming a more interesting question whether it was wise to tie down these popular holidays to the ever-changing times at which Easter and Whitsuntide occur, and whether it would not be better to establish them at fixed dates so arranged as to offer the greatest possible probabilities of decent weather. The Easter holiday itself may not matter so much, everyone expects the weather to be changeable and often cold; but Whitsuntide ought to be an affair of summer—it is a time when hundreds of thousands of children are taken into the country, are tempted to spend their time dancing about in fields, and are brought back by waggon loads long, weary drives in chilly evenings. Many are the attacks of "cold," bronchitis, and pneumonia which date from Whitsuntide excursions, and it would be an untold benefit if the time of these excursions could be pushed on, out of the fickle month of May into sunny June.

It is not a large matter. It merely means fixing Bank Holidays by days of the month instead of by festivals of the Church. It would save every year a large amount of disease and give even a larger amount of health, and it must always be remembered that while the time of this great Easter feast was suitable enough in the countries in which it was first appointed—countries in which the ripening of the barley took place in early March—it is entirely inappropriate for public holidays in northern climes. But there are other reasons why such holidays should be separated from the commemoration of the Churches. So far as Good Friday, Easter Sunday, and Whitsuntide are to be taken as meaning anything in relation to Church history, they can only be taken as commemorative of solemn and serious events. Those people, then, who look upon them as such should welcome any proposal which would put a stop to what to them is an annual outrage on Christianity, viz., the desecration of Good Friday; while those who look more lightly on such matters should be glad of a reform which would give to the people the full benefit, from a health point of view, of two great "Bank Holidays," fixed at such times as to give the greatest probability of fine weather in the place of the present moveable feasts of Easter Monday and Whit Monday.

